# Supplementary material for: Multi-Modality, Multi-Dimensional Characterization of Pediatric Non-Alcoholic Fatty Liver Disease
Source: Metabolites. 2023 Aug 8;13(8):929. doi: 10.3390/metabo13080929 (PMC10456937; doi:10.3390/metabo13080929)

Histopathological Measurement Histograms

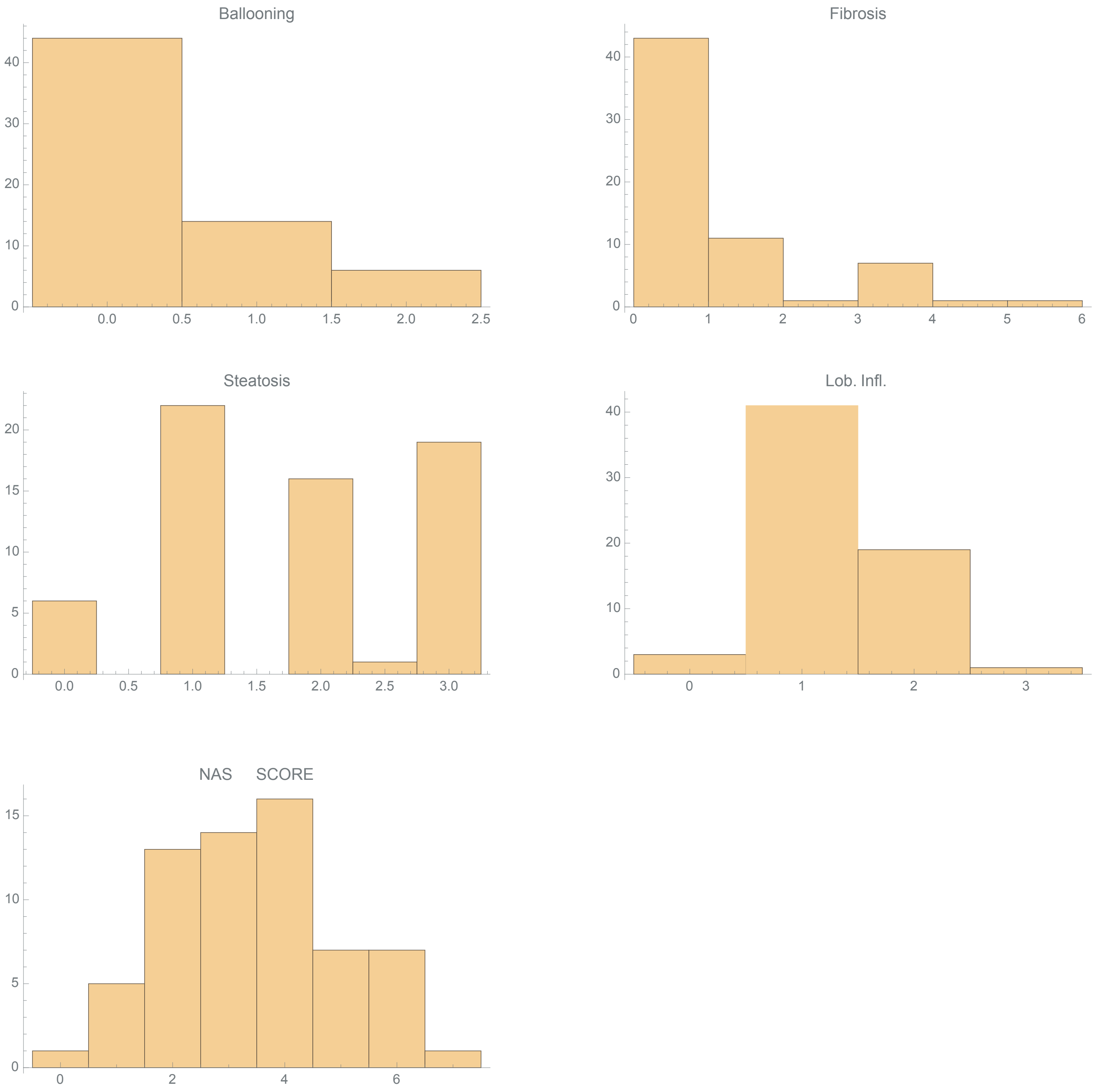

Supplement: Supplementary file 1 [file metabolites-13-00929-s001.zip › supp_figure_S1.pdf]
